# Maternal Vaccination. Immunization of Sows during Pregnancy against ETEC Infections

**DOI:** 10.3390/vaccines5040048

**Published:** 2017-12-06

**Authors:** Jose Matías, Melibea Berzosa, Yadira Pastor, Juan M. Irache, Carlos Gamazo

**Affiliations:** 1 Department of Microbiology, University of Navarra, Instituto de Investigación Sanitaria de Navarra (IDISNA), C/Irunlarrea, 1, 31080 Pamplona, Spain; jmatias@alumni.unav.es (J.M.); mberzosa.1@alumni.unav.es (M.B.); ypastor@alumni.unav.es (Y.P.); 2 Department of Pharmacy and Pharmaceutical Technology, University of Navarra, Instituto de Investigación Sanitaria de Navarra (IDISNA), C/Irunlarrea, 1, 31080 Pamplona, Spain; jmirache@unav.es

**Keywords:** maternal vaccination, pig, ETEC, adjuvant, nanoparticle

## Abstract

The immunology of pregnancy is an evolving consequence of multiple reciprocal interactions between the maternal and the fetal-placental systems. The immune response must warrant the pregnancy outcome (including tolerance to paternal antigens), but at the same time, efficiently respond to pathogenic challenges. Enterotoxigenic *Escherichia coli* (ETEC) strains are a major cause of illness and death in neonatal and recently weaned pigs. This review aims to give an overview of the current rationale on the maternal vaccination strategies for the protection of the newborn pig against ETEC. Newborn piglets are immunodeficient and naturally dependent on the maternal immunity transferred by colostrum for protection—a maternal immunity that can be obtained by vaccinating the sow during pregnancy. Our current knowledge of the interactions between the pathogen strategies, virulence factors, and the host immune system is aiding the better design of vaccination strategies in this particular and challenging host status. Challenges include the need for better induction of immunity at the mucosal level with the appropriate use of adjuvants, able to induce the most appropriate and long-lasting protective immune response. These include nanoparticle-based adjuvants for oral immunization. Experiences can be extrapolated to other species, including humans.

## 1. The Immunodeficient Mother and Child; Just a Matter of Evolution

There is a critical balance in the evolution of the immune system that will be modulated along the whole life of the individual: inflammatory response (e.g., against pathogens) vs. tolerance (e.g., to the individual’s own antigens and the normal microbiota). It is interesting to note how the observed differences in the immune system along the phylogenetic scale are a direct consequence of natural selection (i.e., shaped by evolution), to respond proficiently in that particular environment and circumstances where the animal is living. It is not fortuitous that a mirror of that adaptation in the animal’s development is present in the development of the individual, from the fetus to older ages, adapting the immune response to evolving necessities such as fighting against pathogens, tissue repair, or the constant surveillance against tumor cells. This is particularly relevant during pregnancy, where a well-controlled balance of the immune system is vital to allow survival against infections in opposition to the tolerance to the offspring, including the mother’s ability to transmit protection to the newborn. As will be discussed in this review, an integrated cluster of neuro-immune–endocrine factors co-evolved to cope with these internal (from fetus) and external (from pathogens) antigens. The knowledge of the influence of these circumstances on the modulation of the immune system in both mother and newborn are relevant in the rational design of a successful vaccination schedule.

## 2. Protection by Maternal Immunity

The generalization of pregnancy as a condition of immune suppression and risk is misleading. Because pregnancy represents the most important period for the conservation of the species, the immune system aims to protect the mother from the environment and to prevent damage to the fetus. Thus, it is more appropriate to refer to pregnancy as a unique condition in which maternal immunity is modulated, rather than suppressed. Therefore, pregnancy should not imply more susceptibility to infectious diseases, but a regulation of the immune system which leads to differential responses depending on the stage in which it is studied [1].

In the 1950s, Sir Peter Medawar proposed the allograft paradigm, considering the placenta as an allograft expressing paternal proteins, consequently, mechanisms by which fetuses scape maternal immune surveillance would be necessary. Current evidence suggests that, although there may be an active mechanism preventing a maternal immune response against paternal antigens, the fetus and the maternal immune system establish a cooperative status; thus, the placental immune status and function must receive special attention when studying the maternal–fetal immune interaction [2]. There are significant differences in the maternal–fetal immunity interface between human and swine, although both present remarkable similarities in the immune system [3]. Particularly relevant is the placental structure. In the human species, the structure of the placenta allows an extensive gestational transfer of maternal antibodies to the developing fetus. On the contrary, the six cell layers between the mother and the fetuses in the sow prevent the transfer of maternal antibodies to the fetuses before birth, and fetuses receive antibodies only postnatally through colostrum and milk. In addition, although the pig fetus becomes immunocompetent at about 70 days of gestation, newborn pigs are only able to generate limited T and B cell responses when challenged with pathogens. To circumvent this functional immaturity in the neonatal period, the newborn piglet develops adaptive immune mechanisms provided by the mother [3]. Again, these adaptations come from the selection-evolution process, and help us to understand the interplay among the different physiological structures and processes involved. This review is focused on the pig conditions, but some human data are also included to highlight the differences, offering important lessons regarding maternal immunity.

## 3. Milk-Derived Immunity

As indicated above, in swine there is not an efficient materno-fetal transfer of immunoglobulins via placenta, and fetuses predominantly receive passive immunity postnatally through lactation. Mammals have evolved the mammary glands, dedicated to the synthesis of milk for the newborn, milk being a perfect food and also a great immune support for the immature and susceptible newborn host. Thus, mother’s milk harbors a plethora of immune effector cells as well as fully active antibodies. Colostrum—and to a lesser extent milk—also contains immunosuppressive cytokines such as TGFβ1 and IL-10, which participate in the induction of tolerance to harmless food antigens and symbiotic bacteria. Milk composition differs among species and the moment of lactation. Breast milk harbors mother’s antibodies against numerous pathogens, the concentration of which is higher in the first days of lactation and is decreased throughout lactation [4,5]. In addition, during periods of infection of either the mother or the infant, a dramatic change in the milk composition is observed to readily respond to the challenge. This response has a great applicability in the vaccination of mothers to protect their offspring. In pig serum, IgA is composed of equal parts of monomeric IgA and dimeric IgA, and both fractions predominate in the sow colostrum over the sIgA form (secretory IgA dimeric) [6]. SIgA is essential in the defense of the mucosal membranes to avoid microorganisms’ entrance into the tissues through a process known as immune exclusion. Whereas IgG promotes opsonization and the killing of pathogenic bacteria through the activity of macrophages and neutrophils, sIgA primary acts through a receptor blockade. Besides, sIgA is considered as an anti-inflammatory factor necessary to control the inflammatory responses to dietary components and symbiotic microbiota.

**Figure 1:**
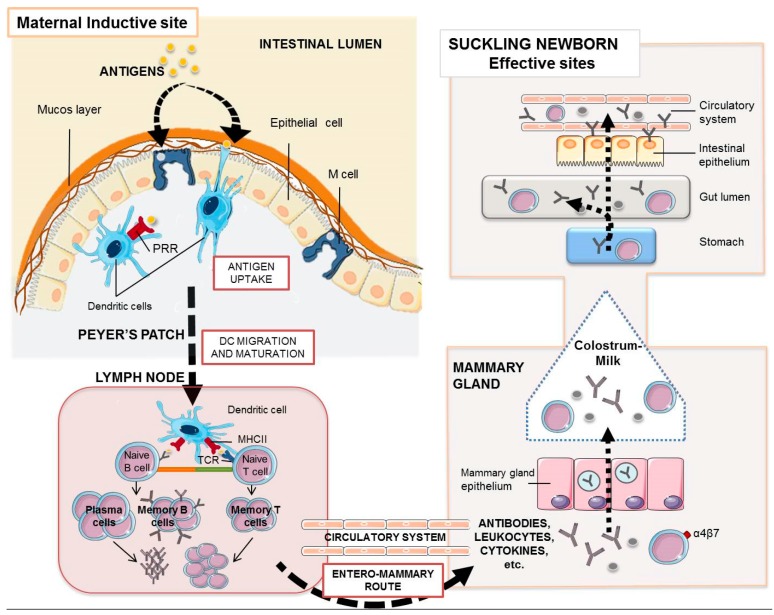
Maternal antibodies and leukocytes in the suckling piglet. The enteromammary route allows the oral maternal immunization to elicit a mucosal and systemic immune response whose humoral and cellular components with effector activity may reach the gut of the piglet. Orally administered antigens, once they reach the intestinal epithelium, are taken up by intestinal microfold (M) cells or by dendritic cells through pattern recognition receptors (PRR), which finally process them and migrate to the proximal lymph nodes where they encounter specific T lymphocytes via TCR (T cell receptors) and B lymphocytes. Activated B cells proliferate and differentiate into antibody-secreting plasm cells. Antibody transfer in pigs is only mediated by colostrum. During these first 2–3 days of life, their enterocytes take up IgG, IgA, or IgM by non-specific endocytosis. After that, the piglet enterocyte facilitates the transport of IgG across the intestinal barrier by the specific FcRn-mediated translocation. Most milk polymeric IgA and IgM stay in the gut lumen for surveillance. Cytokines may also use the “leaky epithelium” around birth to cross the enterocytes to the blood stream. Some plasma cells and maternal lymphocytes present in colostrum are transported to the Peyer’s patches and mesenteric lymph nodes of the suckling piglet.

As mentioned above, antibodies’ decreasing concentration throughout lactation is connected with the change of predominant isotype. Thus, IgG is the predominant immunoglobulin in sow colostrum, and IgA dominates in the mature milk. IgG levels decrease from 98 mg/mL in first colostrum to 4 mg/mL at day 6 of lactation. The concentration of sIgA decreases from 23 to 6 mg/mL, and IgM concentration decreases from 9 to 2 mg/mL at the same time [7,8]. IgG, IgM, and sIgA play important roles in the protection of newborn’s mucosal surfaces [4]. The gut absorption of colostral immunoglobulins in the neonate is mediated by specific (FcRn) and non-specific transport [9]. Once they are absorbed, they may undergo reverse transudation from the neonatal blood through the epithelial cells of the gut [10] and the respiratory tract of piglets [11]. Immune cells are also abundant in milk, particularly in colostrum, and provide transitory immunity to the newborn [12,13,14,15]. The number of leukocytes and lymphocytes recruited to the mammary gland during pregnancy in sows increases significantly from the 80th day of pregnancy, including CD4^+^ and CD8^+^ T cells, B cells, and macrophages. In milk, macrophages represent 5–10% of the immune cells, similar to the levels of lymphocytes, T cells being the most prominent (>80%). Specifically, T CD8^+^ cells express L-selectin, α4β7 integrin, and mucosal addressin cell adhesion molecule-1; and T CD4^+^ cells express activation markers CD40L, sCD30, IL-2 receptor, human mucosa lymphocyte antigen-1, or late activation protein-1 and CD45RO^+^ (Figure 1). It has been hypothesized that activated T cells compensate the immature function of neonatal T cells and promote their maturation. Moreover, activated antigen mature lymphocytes might help to compensate the low antigen-presenting capacity of macrophages [4,16].

## 4. Maternal Antibodies and Leukocytes in the Suckling Infant

Antibodies’ transference across the placenta depends on placenta structure and the route of antibody translocation. Humans have a hemochorial placenta and IgG transfer is mediated by FcRn, whereas pigs have an epitheliochorial placenta which prevents intra-uterine passage of antibodies from mother to fetus. This type of placenta has six cell layers, and inhibits the passage of immunoglobulins and other immunological factors to the fetus during pregnancy. Consequently, IgG transfer in pigs is only mediated by colostrum [17,18,19,20,21]. Thus, the piglet enterocyte has evolved to facilitate the transport of IgG from colostrum across the intestinal barrier to reach the systemic circulation; a non-specific endocytosis occurs during the first 2–3 days, and after that, an FcRn-mediated translocation. Along those first 2–3 days of life, the piglet enterocytes could take up IgG, but also IgA or IgM, until “gut closure” [5,19,22]. After that, the door is open through specific endocytosis. The porcine FcRn is expressed on the luminal apical surface of gut epithelial cells in suckling neonates, but also in adult animals. This adaptation has important consequences in immune surveillance, since this receptor is involved in the import and export of IgG. In the neonate, FcRn allows the ingested maternal IgG to be taken up from the gut lumen into the blood (see above, passive immunity). In the adult, the IgG produced in the intestinal Peyer’s patches uses this route to be released to the gut lumen as part of the mucosal defense [20,23]. After “gut closure”, milk polymeric IgA and IgM use the pIgR (polymeric immunoglobulin receptor) to cross the epithelial cells and reach the blood stream, or may stay in the gut lumen for surveillance. pIgR is located at the basolateral allowing unidirectional transport of polymeric antibodies into the lumen. However, some intriguing results suggest a bidirectional trafficking of polymeric IgA [11,24].

Perinatally, cytokines may also use the “leaky epithelium” to cross into the blood stream [12,25,26] until the gut closure. TNF-α is the only maternal cytokine not found in piglets [27].

A high number of leukocytes are present in the milk, reaching and crossing the infant´s intestinal epithelium. This is a consequence of the low stringent environmental conditions found in the newborn stomach during breast-feeding; however, our knowledge on the mechanisms of translocation is still limited. Maternal γδ lymphocytes are frequently in colostrum, and have been detected in suckling piglets. These cells are known to be transported to the mesenteric lymph nodes [26] and to other tissues [12], where they promote an immune response to unprocessed MHC-unrestricted antigens [12,28]. Functional cytotoxic specific CD8 T cells expressing the gut homing markers α4β7 and CCR9 have also been found in milk and in the Peyer’s patches of the suckling infants, as well as B and plasma cells (see below, section *Targeting the Mucosal Immune System*) [29,30]. Finally, milk contains large amounts of myeloid cells (e.g., macrophages and granulocytes), but the transference to the infant’s intestine during suckling and its physiological relevance is unknown [30,31].

## 5. Acquired Specific Piglet Immunity through the Sow

The pork-processing industry is being considered as the fastest growing sector of the food industry. In fact, more pork is eaten in the world than any other meat—specifically, over a third of the consumed meat [32]. Unfortunately, the increase in animal production is correlated with the emergence of novel pig-borne pathogens, some of them with significant zoonotic potential [33,34]. Significantly, *Escherichia coli* infections are the most important causes of disease in pigs. There are several pathotypes of *E. coli* causing enteritis (enterotoxigenic (ETEC); vero- or Shiga-like toxin producing (VTEC or STEC); nacrotoxigenic (NTEC); enteropathogenic (EPEC); enterohaemorrhagic (EHEC); enteroaggregative (EAggEC); and enteroinvasive (EIEC)) with different pathogenicity, epidemiological, and clinical courses [35]. Specifically, ETEC serotypes produce the highest rates of morbidity and mortality during neonatal and post-weaning periods [36,37,38,39].

As discussed above, neonatal ETEC infections can be prevented by lactogenic immunity obtained by vaccination of the sow. In this respect, several maternal vaccines are on the market with different vaccine approaches, including the use of bacterins and subunit antigens (fimbriae, toxoids), and in some cases, multivalent vaccines against common diarrhea-causing pathogens (Table 1). Their common goal is to elicit the production of specific antibodies against main adhesion factors and toxins of ETEC strains in the colostrum and milk of sows to prevent the mortality of piglets [40,41,42]. In addition, other virulence factors are also being experimentally studied as potential components of vaccines against ETEC [43]. These findings may have significant implications for the development of vaccines against ETEC.

**Figure 2:**
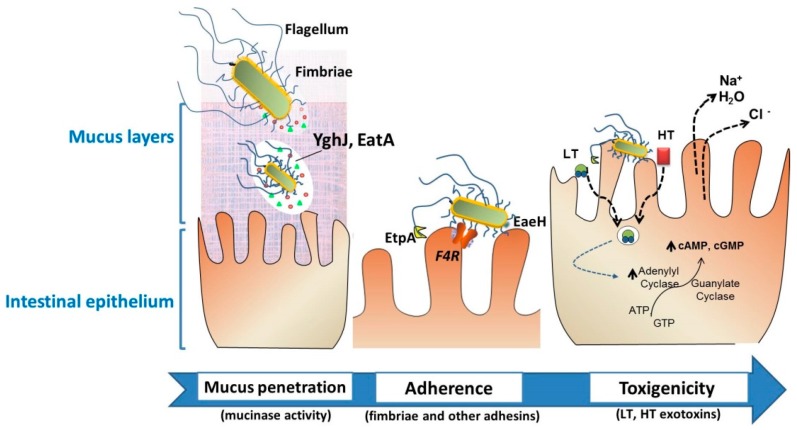
Intestinal colonization through the action of particular virulence factors of enterotoxigenic *Escherichia coli* (ETEC). Neonatal and post-weaning pig diarrheas are mostly associated with the colonization of F4+. To effectively deliver the exotoxins, the bacteria must traverse the protective layer of mucin in the intestinal lumen, and engage with the epithelial cell. EatA and YghJ are ETEC proteins capable of degrading intestinal mucins. Bacterial adhesion involves F4 fimbriae through the F4-receptor, flagella, secreted proteins such as EtpA, or the surface-exposed EaeH protein that support the subsequent intimate connection with the enterocyte. The delivery of heat-labile toxins (LT) and heat-stable toxin (ST) brings the release of electrolytes and water, leading to severe watery diarrhea. These ETEC-associated factors—among other virulence factors—are considered putative targets for vaccine development.

Layers of mucin glycoproteins act as a major barrier to ETEC interaction with the epithelial surface [44]. EatA and YghJ are mucin-degrading enzymes released by ETEC strains to reduce the viscosity of the mucus layer (Figure 2). Studies performed in mice demonstrate that vaccination with EatA afforded significant protection against infection [45,46] Moreover, YghJ is recognized by convalescent antibody following ETEC infection [47].

Flagellar motility is important to resist peristalsis and colonize the small intestine. ETEC strains are peritrichous, and each flagellum contains over 20,000 flagellin protein molecules that mediate adhesion to enterocytes. Vaccination with flagellin generates antibodies that afford significant protection against ETEC in experimental mouse models [48]. Besides, it has been shown that efficient adherence of ETEC to intestinal cells requires both intact flagella but also EtpA—a secreted adhesin that mediates the indirect adhesion of flagellin to receptors on the enterocytes. In this sense, the immunization with recombinant EtpA was able to inhibit ETEC colonization in mice [48,49]. Many other colonization factors—including fimbriae—have been identified in ETEC isolates which mediate adhesion to specific receptors on the small intestinal enterocytes, resulting in a morphologically non-destructive attachment of bacteria to the microvilli (Figure 2). Furthermore, nonclassical adhesins may have special relevance during colonization; an example is EaeH, an outer membrane protein adhesin required at a later step in ETEC–host interactions [50] that has also been identified as an immunogenic protein with vaccine potential [49]. The *F4* and *F18* fimbrial serotypes are the most prevalent in post-weaning diarrhea (PWD) by ETEC [37], and consequently, these adhesins are present in most commercial vaccines (Table 1). F4 fimbriae are composed of a major subunit (FaeG) and minor subunits (FaeC, FaeF, FaeH, FaeI, and FaeJ), and specifically interact with the F4R receptor on their intestinal epithelial cells (Figure 2) [51]. Purified ETEC F4 fimbriae were immunogenic after oral administration in weaned piglets [52].

After colonization, the bacteria secrete enterotoxins which include heat-labile toxins (LT) and heat-stable toxin (ST) (Figure 2). LT toxins are transported by the type-2 secretion system through the bacterial outer membrane, remains associated to the lipopolysaccharide (LPS), and it is further secreted into vesicles that are released from the outer membrane. These LT-decorated vesicles bind to the enterocytes, allowing the LT to reach the cytosol, and consequently activates adenylate and guanylyl cyclases, which increases the intracellular concentration of cyclic adenosine monophosphate. These factors alter the functions of enterocytes by increasing water and electrolyte secretion and reducing absorption, resulting in osmotic diarrhea. ST is directly released upon bacterial adherence and activates guanylyl cyclases (Figure 2) [53]. Accordingly, for protection against ETEC diarrhea, specific antibodies that inhibit bacterial interaction to the intestinal cells and/or neutralize enterotoxins are essential.

## 6. Maternal Vaccines. Targeting the Mucosal Immune System

As indicated before, newborn and weaned animals are extremely susceptible to ETEC infections due to the lack of protection at birth. During this time, resistance to infection depends mainly on the actions of the innate defense mechanisms and specific antibodies transferred passively from sow to piglet through colostrum and milk [54]. This maternally-derived immunity must provide sufficient protection during the period in which the piglet gradually develops its own active immunity. For this purpose, the challenging goal is to use vaccine formulations which are able to induce a strong mucosal immune response. However, when vaccines are administered parenterally, they generally stimulate a systemic rather than a mucosal immune response and, paradoxically, most of the maternal vaccines on the market are applied parenterally.

The vaccination route is a critical factor to induce the right immunity at the right site. The route of administration has a great influence on the expression of chemokine receptors, selectin ligands, and homing factors that dictate the migratory properties of activated T cells toward the specific sites of infection. Targeting the mucosal immune system, for instance, is essential against enteric infections where gut mucosa is the first barrier in the defense against such pathogens. The best scenario for the host is to detain the pathogen in the portal of entry at the mucosae before it gains entry into the body and starts massive colonization and invasion. Therefore, in order to develop a vaccine which is capable of controlling ETEC infections, it would be necessary to stimulate the specific mucosal immune system at the intestinal level [55]. Another factor to be considered is that the mucosal immune system is integrated as a network, named as the “common” mucosal immune system. This refers to the evolved ability to induce immune responses on distal regions of the systemic and mucosal immune systems from the original site of antigen inoculation. This property has a tremendous practical applicability. Among the different mucosal routes of vaccination, the oral route seems to be the preferred one due to its safety (needle-free) and ease of administration (painless). Despite this fact, few oral vaccines are currently commercialized due to the difficulties that must face through the gastro-intestinal system. First, oral vaccines must be able to successfully reach the intestine after passing through the extreme acidic environment of the stomach, and resist the physico-chemical barriers found in the intestine, such as antibacterial proteins, digestive enzymes, and the peristaltic movements. Even more, once they reach the intestinal epithelium, they must interact with antigen-presenting cells (APCs) which are present in gut-associated lymphoid tissue (GALT) [44]. The arrival of antigens to GALT is not an easy task due to the complex matrix that constitutes the mucus barrier composed of several joined layers of lipids, salts, and mucins, tightly attached to each other and to the internal glycocalyx layer [56]. Antigens capable of making it successfully are taken up by intestinal microfold (M) cells or by dendritic cells (DCs) (Figure 1), which finally process them and migrate to the proximal lymph nodes where they encounter specific T and B lymphocytes. Activated B and T cells proliferate and differentiate, leaving germinal centers and entering the systemic circulation to trigger a specialized immune response. Most activated circulating lymphocytes home back to site where the antigen was initially encountered, whereas some others go to other distal mucosae, including the mammary glands [57].

The mammary glands have evolved as an anatomical and functional extension of the mucosal immune system of the gut, and consequently, the oral immunization of the mother will influence the immunity of the suckling offspring. As indicated in the previous section, milk contains a large variety of leukocytes [30]. Lymphocytes with the homing integrin α4β7 migrate from the mother’s intestinal PPs to the lactating mammary gland thanks to the highly-expressed addressing MadCAM-1 (α4β7 ligand). Similarly, when lactation begins, plasma cells from the intestinal PPs migrate to the mammary gland. These cells release the dimeric IgA, which will traverse through the mammary epithelial cells via the IgA receptor to be excreted in the milk. B cells are also present in the mammary gland, but B cell numbers increase when T cells decrease; this could be related to the different migration pathways of B and T lymphocytes determined by the different expression of homing markers and addressins [58]. In sum, these immune adaptations support the maternal immunization by oral route to protect the neonate. However, there is the major problem that oral vaccines have to face tolerance. To avoid a permanent “inflammation”, the mucosal tissues exposed to environmental antigens (e.g., food antigens) present a tolerogenic tendency. To break tolerance, the host must “sense” danger signals. It is therefore critical that new vaccine candidates contain not only the right immunogens, but also danger signals such as pathogen-associated molecular patterns (PAMPs), present either in the antigenic complex, in the adjuvant, or in both [59,60].

## 7. Different Types of Vaccines

There are several types of vaccines attending to their antigenic nature. Attenuated vaccines consist of living pathogens; so, they do contain PAMPs, and consequently, with a low dose evoke a high and sustainable immune response which is both cellular and antibody response-mediated. However, they might still present residual virulence, and could theoretically revert to virulent forms or may multiply and cause disease in some particular immunocompromised population (e.g., mothers and newborns), and moreover, even may pass the placental barrier to the fetus. Therefore, suitable precautions must be taken it its use. [61,62]. Coliprotec^®^ F4 (Prevtec Microbia GmbH, Montrela, Canada) [63] and the recently reported oral bivalent F4/F18 vaccine [64] are attenuated vaccines designed for the active immunization of pigs against PWD, but there are no data available on their effect in sows.

Inactivated vaccines contain pathogens that have been inactivated with chemical or physical procedures, which makes these vaccines safer for the host compared to attenuated vaccines. A commercial vaccine manufactured by Elanco is composed of a mixture of four ETEC bacterins (F4, F5, F6, and F41 strains), and the commercial vaccine developed by Vencofarma contains a mixture of bacterins from F4, F5, F41, and 987P strains: (Table 1). These vaccines may be administered to pregnant pigs two weeks before farrowing, but need to be applied parenterally [54,65].

On the other hand, subunit vaccines contain raw or purified antigens, or even nucleic acids coding antigens of the pathogen. The risk of adverse effects decreases, but are less immunogenic and often require the use of adjuvants. As indicated above, ETEC strains are non-invasive, and therefore vaccines must contain main toxoids and main adhesins. Toxoid vaccines are highly stable, but tend not to be very immunogenic, and then—even with adjuvants—require booster doses to elicit an appropriate memory immune response [66,67]. Several groups are using the LT_192_ toxoid—a non-toxic mutant of the heat-labile enterotoxin [68]. The genetic fusion of LT_192_ with ST toxoid enhanced anti-ST immunogenicity and elicited protective anti-LT and anti-ST immunity [69]. Commercial vaccines for sows, such as Porcilis-coli^®^ and Suiseng^®^, include the LT toxoid [54] and also contain fimbriae (Table 1). However, they require a parenteral administration, and passive lactogenic protection is rapidly lost after weaning.

Oral vaccination implies a sustainable, practical, and effective approach to obtaining long-lasting lactogenic immunity. The gut-associated lymphoid tissues contain the largest pool of immunocompetent cells in the body, including dendritic cells, B cells, plasma cells, subsets of CD4^+^ T cells, and even CD8^+^ T cells. Thus, high and durable immunity can be achieved through oral vaccination against mucosa-associated pathogens such as *E. coli*. However, there are some drawbacks that need to be considered. Limitations of the effective oral delivery of vaccines reside in various factors: antigenic components must be resistant to the hostile gastric and intestinal environments; they must resist peristalsis and be able to penetrate the dense mucous layer; and finally, adhere and transcytose the epithelial intestinal cells. Therefore, many vaccine formulas remain ineffective because of their poor bioavailablity when administrated orally. To protect orally-delivered antigens against capturing by lactogenic antibodies and chemical or enzymatic gastrointestinal degradation, coated pellets have been used [70]. The use of F4 fimbriae in enteric-coated pellets was compared to F4 fimbriae in solution in orally vaccinated suckling pigs. The enteric-coated pellets released the F4 fimbriae in the beginning of the jejunum, near the jejunal Peyer’s patches, allowing them to bind to specific receptors present on enterocytes and/or M cells, and resulted in a marginal but significant reduction in the excretion of the F4 strain upon challenge. This was not observed when suckling pigs were orally vaccinated with F4 fimbriae in solution, demonstrating that protection of the antigen against degradation and inactivation by enzymes in the stomach and the beginning of the small intestine and/or against neutralization by milk factors has beneficial effects [70]. However, this approach has not been tested in sows, since as stated previously, a strong “danger” signal is required to breach the tolerogenic status of the pregnant animal. Polymeric nanoparticulate delivery systems (NPs) are well-recognized adjuvants suitable to overcome all the barriers that still challenge oral vaccination [44,71,72]. The rationale of mucosal vaccination using antigens loaded or encapsulated into nanoparticles is based, firstly, on the protection of an antigen from exposure to extreme pH conditions, bile, and pancreatic secretions; second, on the interaction with different components of the mucosa and the ability to go through the mucosal layers and reach the epithelium; and, third, the inherent inclination of submicron particles to be naturally captured by M cells and antigen-presenting cells as part of their duties as sentinels in triggering mucosal immunity against pathogens [44,73].

NPs are polymeric particles with a typical size of 200 nm. Size also has a significant influence on the cellular uptake and type of immune responses induced in the gut. Another important factor of NPs as adjuvants is related to the nature of the polymer used to produce them. The polymer determines the stability of the resulting particles in the gastrointestinal tract, as well as their interaction with components of the mucosa. Furthermore, the adequate selection of the polymer deeply determines the final antigen loading in the resulting nanoparticles. Examples of polymers likely to be used to produce nanoparticles with a full maintenance of structural and antigenic conservation include: poly(d,l-lactide-*co*-glycolide) (PLGA), copolymers between methyl vinyl ether and maleic anhydride (PVM/MA) (Gantrez^®^, Ashland, NJ, USA), cationic cross-linked polysaccharides chitosan, lipids, starch, phosphazene, poly(epsilon caprolactone), or cationic cross-linked polysaccharides [74,75,76,77]. Felder et al. encapsulated F18 fimbriae in PLGA microparticles and used it to orally inoculate weaned pigs. However, specific antibodies were not detected [78]. Interestingly, some poly (anhydride) nanoparticulate systems made by the copolymers of methyl vinyl ether and maleic anhydride (PVM/MA) have demonstrated their efficacy as adjuvants to induce Th1 immune responses [79,80]. These NP formulations induced innate immune responses mediated in a TLR-2 and TLR-4 dependent manner. This is an important finding since, as it was indicated above, it has been shown that the use of multiple PAMPs influences the induction of long-term memory cells, the ultimate goal for any vaccine being the stimulation of long-lasting protective immunological memory. Vandamme and co. tested the adjuvanticity of methylvinylether-comaleic anhydride (Gantrez^®^) on oral delivery of F4 fimbriae to weaned pigs. Encapsulation of F4 in Gantrez^®^ nanoparticles raised the serum antibody response against F4, but did not improve protection as compared to soluble F4 fimbriae. Moreover, the best effect was observed when empty nanoparticles were added to soluble F4 fimbriae, suggesting that adjuvant properties rather than protection of the antigen against gastrointestinal degradation were responsible for the enhanced antibody response [81]. Another strategy to render nanoparticles more efficient as adjuvants for oral vaccination consists of their association with microbial adhesins including lipoteichoic acids, outer membrane proteins, flagellum, fimbriae lectins, and glycoproteins (i.e., mannosamine). Thus, it has been demonstrated that the association of either flagellin from *Salmonella enterica* flagellum or mannosamine to Gantrez AN nanoparticles could enhance the bioadhesive capabilities of the resulted decorated nanoparticles. These nanoparticles demonstrated a high ability to colonize the gut of animals, particularly the ileum, and high affinity to Peyer’s patches [82]. Using ovalbumin as model antigen, “*Salmonella*-like” nanoparticles induced a strong and balanced secretion of both IgG2a (Th1) and IgG1 (Th2) specific antibodies. In addition, these nanoparticles were able to induce a stronger mucosal IgA response than control nanoparticles. Recently, the same group has developed a new formulation based on nanoparticles found to be safe after oral administration of pregnant sows. This new vaccine formulation consists of purified outer membrane vesicles from F4 and F18 strains encapsulated in zein-based nanoparticles, and was able to elicit a potent mucosal maternal immune response that was passively transferred to the suckling piglets [83].

## 8. Final Remarks

The maternal administration of a vaccine needs to be safe; the production of humoral and cellular immune effector components after oral vaccination is possible; the newborn gut status allows the tolerance and absorption of those maternal factors. However, the challenge is to get the right maternal induced immunogenicity to be protective in the suckling offspring. Our knowledge of the pathogenicity of ETEC strains allows us to design vaccines with the potentially correct antigens from the main virulence factors responsible for the bacterial virulence. Thus, the success of maternal immunization and transferences of immunity to neonates will rely on the integration of knowledge of the pathogen virulence factors, the particular host immune system of both mother and offspring, and the new advances in adjuvant design. In fact, a potent and persistent presence of mucosal immune effector components is required in order to arrest the pathogen at the portal of entry. New adjuvants can be used to reach these goals, to protect mother and fetus during pregnancy, and to elicit a proper immune response [84]. Some polymeric nanoparticles have been demonstrated to be safe and potent oral adjuvants. However, as mentioned above, the projection of any formulation to be applied in the pregnant target is not straightforward. Large animal trials are needed to determine the protective efficacy of these new maternal vaccine approaches.

## Figures and Tables

**Table 1:** Vaccines available for use in sows against ETEC.

Vaccine	Composition	Route	Adjuvant	Weeks before Farrowing	Manufacturer
Porcilis^®^ coli	LT toxoid	Parenteral		Unvaccinated gilts and sows	MSD Animal Health (Kenilworth, NJ, USA)
	Fimbriae (F4ab, F4ac, F5, F6)			1st dose: 6–8 weeks 2nd dose: in the second half of pregnancy	
Porcilis^®^ 2 * 4 * 3	- ETEC bacterins: K88, K99, 987P, F4.	Parenteral		Unvaccinated gilts and sows	MSD Animal Health (Kenilworth, NJ, USA)
	- Inactivated *Clostridium perfringens* type C			1st dose: 6–8 weeks 2nd dose: in the second half of pregnancy	
Suiseng^®^	- LT toxoid	Parenteral		Unvaccinated gilts	HIPRA (Gerona, Spain)
	Fimbriae (F4ab, F4ac, F5, F6)			1st dose: 6 weeks	
	- β-toxoid of *Clostridium perfringens* type C.			2nd dose: 3 weeks	
				Sows	
				One dose: 2–3 weeks	
PILI SHIELD^®^	ETEC bacterins (K88, K99, 987P, F41 strains).	Parenteral		Unvaccinated gilts	Elanco (Greenfiled, IND, USA)
				1st dose: 5 weeks	
				2nd dose: 2 weeks	
				Sows	
				One dose: 2 weeks before delivery	
SERKEL GASTRO RV^®^	- ETEC bacterins: (K88, 987P, K99, F41)	Parenteral		Unvaccinated gilts	Vencofarma (Paraná, Brazil)
	- Inactivated Rotavirus			1st dose: 5 weeks	
	- Toxoids from *Clostridium perfringens* type C and D.			2nd dose: 2 weeks	
				Sows	
				One dose: 2 weeks	
Clostricol	- *Escherichia coli* bacterins K87, K88; O149: K91, K88; O101: K (A, K99, 987p.	Subcutaneous	Aluminium hydroxide	Sows	IDT Biologika GmbH (Dessau-Roßlau, Germany)
	- *Clostridium perfringens* type C toxoid.			1st dose: 5 weeks 2nd dose 2 weeks	
Colidex-C	- *Escherichia coli* bacterins K88, K99, F41, F18, P987.	Parenteral	Mineral oil	Unvaccinated gilts	CZ Veterinaria S.A. (Porriño, Spain)
	- *Clostridium perfringens* type C toxoid.			1st dose: 7 weeks	
				2nd dose 4 weeks	
				Revaccinated Sows	
				One dose: 4 weeks	
Coliporc PLUS	*Escherichia coli* bacterins O8; K87, K88 (F4); O149: K91, K88; O101 K99.	Subcutaneous	Aluminium hydroxide	Sows	IDT Biologika GmbH (Dessau-Roßlau, Germany)
				1st dose: 5 weeks 2nd dose: 2 weeks	
Colisuin-CL	- *Escherichia coli* fimbriae: 987P, K88ab, K88ac, K99	Parenteral	Oil adjuvant	Unvaccinated gilts	HIPRA (Gerona, Spain)
	- *Clostridium perfringens* type C toxoid.			- 1st dose: 8 weeks	
	- *Clostridium novyi* toxoid.			- 2nd dose: 4 weeks	
				Sows	
				One dose: 4 weeks	
Colisuin-TP	*Escherichia coli* fimbriae: 987P, K88ab, K88ac, K99.	Parenteral	Liquid paraffin, Montanide 888	Unvaccinated gilts	HIPRA (Gerona, Spain)
				- 1st dose: 8 weeks	
				- 2nd dose: 4 weeks	
				Sows	
				One dose: 4 weeks	
Combined Gastroenteritis, Rotavirus and *E. coli*	- Inactivated Rotavirus	Intranasal, intramuscular	Oil emulsion	The emulsified vaccine is administered twice: on the 5–6 weeks and 2–3 weeks.	Narvac (Moscow, Russia)
	- *Escherichia coli* somatic 09, 078, 0141; capsular polysaccharides K80, K30, K87, K88			- 1st dose: 13–14 weeks	
				- 2nd dose: 10 weeks	
				The dry vaccine is administered together with the emulsified one 10 weeks	
ECOvac *E. coli*	*Escherichia coli* bacterins: K88, K99, 987P	Intramuscular		Unvaccinated gilts	MSD Animal Health (Kenilworth, NJ, USA)
				- 1st dose: 7 weeks	
				- 2nd dose: 3 weeks	
				Sows	
				One dose: 3 weeks	
Combined ECOvacLE	- *Escherichia coli* bacterins K88, K99, 987P.	Parenteral		Unvaccinated gilts	MSD Animal Health (Kenilworth, NJ, USA)
	- *Leptospira interrogans* bacterin.			- 1st dose: at selection	
	- *Erysipelothrix rhusiopathiae* bacterin			- 2nd dose: 4–6 weeks later	
				- 3rd: 3 weeks	
				Sows with unknown vaccination history:	
				- two vaccinations 4–6 weeks apart.	
				Revaccination	
				- booster dose at 3 weeks	
Kolierysin NEO	- *Escherichia coli* bacterins O147:K88 (F4) ab, O149:K88 (F4) ac, O101:K99 (F5), 987P (F6) and O101:K99:F41.	Parenteral	Oil emulsion	Sows and gilts	Bioveta, A.S. (Ivanovice na Hané, Czech Republic)
	- LT toxoid			- not later than 5 weeks	
				Revaccination with the single dose of the vaccine KOLISIN NEO: 10–14 days later;	
				repeated 2–3 weeks before each next expected delivery.	
Kolisin NEO	- *Escherichia coli* bacterin O147:K88 (F4) ab, O149:K88 (F4) ac, O101:K99 (F5), 987P (F6) and O101:K99:F41.	Parenteral	Oil emulsion	Sows and gilts	Bioveta, A.S. (Ivanovice na Hané, Czech Republic)
	- LT toxoid			- not later than 5 weeks	
				Revaccination with the single dose of the vaccine KOLISIN NEO: 10–14 days later;	
				repeated 2–3 weeks before each next expected delivery.	
LitterGuard	*Escherichia coli* bacterins K99, K88, 987P, F41	Parenteral		Primary vaccination:	Zoetis [Pfizer; Fort Dodge Animal Health] (Gerona-Spain)
				- 1st dose: 2 weeks	
				- 2nd dose: 2 weeks	
				Revaccination:	
				- One dose: 2 weeks before each subsequent farrowing.	
LitterGuard LT-C	- *Escherichia coli* bacterin K99, K88, 987P, F41.	Parenteral		Primary vaccination:	Zoetis [Pfizer; Fort Dodge Animal Health] (Gerona-Spain)
	- *Clostridium perfringens* type C toxoid			- 1st dose: 4 weeks	
				- 2nd dose: 2 weeks	
				Revaccination:	
				One dose: 2 weeks before each subsequent farrowing	
Neocolipor	*Escherichia coli* fimbriae: F4 (F4ab, F4ac, F4ad), F5. F6. F41.	Parenteral	Aluminium hydroxide	Primary vaccination:	Boehringer Ingelheim (Duluth, Georgia, USA)
				- 1st dose: 5–7 weeks	
				- 2nd dose: 2 weeks	
Neumosan	- *Escherichia coli* bacterin K99	Subcutaneous	Aluminium hydroxide	Primary vaccination:	Laboratorios Santa Elena S.A. [Virbac] (Montevideo, Uruguay)
	- *Mannheimia haemolytica* bacterin			two doses with an interval of 3–4 weeks	
	- *Pasteurella multocida* bacterin			Revaccinate annually.	
	- *Salmonella enterica* Dublin bacterin				
Polyvalent colibacteriosis	*Escherichia coli* bacterins 06, 09, 0138, 0139, 076, 0141, 0147, 0149 and K88 (optional)	Intramuscular	Aluminium hydroxide	6–8 weeks	Diavak (Radovljica, Slovenia)
Porcine *E. coli* vaccine—Polyvalent	*Escherichia coli* bacterins K88, K99, 987P, F41.	Subcutaneous	Aluminium hydroxide gel	- 1st dose: 5–6 weeks	Green Cross Veterinary Products Co. Ltd. (Chungcheongnam-do, Korea)
				- 2nd dose: 2–3 weeks	
Prefarrow Shield 9	- *Escherichia coli* bacterins K88, K99, 987P, F41.	Intramuscular		Sows and gilts:	Elanco (Greenfiled, IND, USA)
	- *Clostridium perfringens* type C bacterin.			- 1st dose: 5 weeks	
	- *Bordetella bronchiseptica* bacterin.			- 2nd dose: 2 weeks	
	- *Pasteurella multocida* type C and D bacterin			Subsequent farrowing:	
	- *Erysipelothrix rhusiopathiae* bacterin			One single dose	
ProSystem RCE	- *Clostridium perfringens* type C bacterin.	Parenteral		Primary vaccination:	MSD Animal Health (Kenilworth, NJ, USA)
	- *Escherichia coli* bacterins K88, K99, 987P, F41.			- 1st dose: 5 weeks	
	- Porcine rotavirus attenuated.			- 2nd dose: 2 weeks	
				In subsequent farrowings:	
				One dose 2 weeks before farrowing.	
Rokovac NEO	*Escherichia coli* O101:K99 (F5); O147:K88 (F4); O149:K88 (F4); K85:987P (F6);O101:K99:F41 (F5, F41)	Parenteral	Oil emulsion	Primary vaccination:	Bioveta, A.S. (Ivanovice na Hané, Czech Republic)
				- 1st dose: 4 weeks	
				- 2nd dose: 2 weeks	
				Revaccination:	
				One dose 4–2 weeks prior to any other expected labor.	
Scourmune-C	- *Escherichia coli* bacterins K88, K99, 987P, F41.	Parenteral	Aluminium hydroxide	Primary vaccination:	MSD Animal Health (Kenilworth, NJ, USA)
	- *Clostridium perfringens* type C bacterin.			- 1st dose: 6–7 weeks	
				- 2nd dose: 3–4 weeks	
				Subsequent farrowings:	
				- one single dose 2–3 weeks prior to each subsequent farrowing.	
Suiven	- *Escherichia coli* bacterins K88, K99, 987P, F41.	Subcutaneous	Aluminum hydroxide gel	4 weeks.	Vencofarma (Paraná, Brazil)
	- *Bordetella bronchiseptic* bacterin.				
	- *Erysipelothrix rhusiopathiae* bacterin.				
	- *Pasteurella multocida* type A and D bacterins.				
	- *Salmonella enterica* bacterin				
	- *Leptospira interrogans* bacterin				
Anaerobic Enterotoxaemia and *E. coli*	- *Escherichia coli* bacterins 08, 09, 0138, 0139, 078, 0141, 0147, 0149, K88, K99.	Intramuscular	Aluminium hydroxide	- 1st dose: 5 weeks	FGUP Armavirskaja (Krasnodarskij Russia)
	- *Clostridium perfringens* type C bacterin.			- 2nd dose: 3 weeks	
*E. coli* Inactivated	*Escherichia coli* bacterins KMIEV-40A, KMIEV-38, KMIEV-98, KMIEV-18 and K88, K99, F41, O18.	Intramuscular	Emulsified oil adjuvant	- 1st dose: 5–7 weeks	Institute for Experimental Veterinary-Medicine, (Kosice, Slovakia)
				- 2nd dose: 2–3 weeks	

## References

[1] Mor G., Cardenas I. (2010). The immune system in pregnancy: A unique complexity. Am. J. Reprod. Immunol..

[2] Racicot K., Kwon J.Y., Aldo P., Silasi M., Mor G. (2014). Understanding the complexity of the immune system during pregnancy. Am. J. Reprod. Immunol..

[3] Butler J.E., Sinkora M., Wertz N., Holtmeier W., Lemke C.D. (2006). Development of the neonatal B and T cell repertoire in swine: Implications for comparative and veterinary immunology. Vet. Res..

[4] Hanson L.A., Korotkova M., Lundin S., Håversen L., Silfverdal S.A., Mattsby-Baltzer I., Strandvik B., Telemo E. (2003). The transfer of immunity from mother to child. Ann. N. Y. Acad. Sci..

[5] Rooke J.A., Bland I.M. (2002). The acquisition of passive immunity in the new-born piglet. Livest. Prod. Sci..

[6] Vaerman J.P., Langendries A., Pabst R., Rothkotter H.J. (1997). Contribution of serum IgA to intestinal lymph IgA, and vice versa, in minipigs. Vet. Immunol. Immunopathol..

[7] Markowska-Daniel I., Pomorska-mól M., Pejsak Z. (2010). Dynamic changes of immunoglobulin concentrations in pig colostrum and serum around parturition. Pol. J. Vet. Sci..

[8] Markowska-Daniel I., Pomorska-mól M. (2010). Shifts in immunoglobulins levels in the porcine mammary secretions during whole lactation period. Bull. Vet. Inst. Pulawy..

[9] Pacha J. (2000). Development of intestinal transport function in mammals. Physiol. Rev..

[10] Ward L.A., Rich E.D., Besser T.E. (1996). Role of maternally derived circulating antibodies in protection of neonatal swine against porcine group a rotavirus. J. Infect. Dis..

[11] Bradley P.A., Bourne F.J., Brown P.J. (1976). The respiratory tract immune system in the pig. Vet. Pathol..

[12] Williams P.P. (1993). Immunomodulating effects of intestinal absorbed maternal colostral leukocytes by neonatal pigs. Can. J. Vet. Res..

[13] Schollenberger A., Degorski A., Frymus T., Schollenberger A. (1986). Cells of sow mammary secretions. I. Morphology and differential counts during lactation. Zentralbl. Veterinarmed. A.

[14] Schollenberger A., Frymus T., Degorski A., Schollenberger A. (1986). Cells of sow mammary secretions. II. Characterization of lymphocyte populations. Zentralbl. Veterinarmed. A.

[15] Salmon H., Berri M., Gerdts V., Meurens F. (2009). Humoral and cellular factors of maternal immunity in swine. Dev. Comp. Immunol..

[16] Eglinton B.A., Roberton D.M., Cummins A.G. (1994). Phenotype of T cells, their soluble receptor levels, and cytokine profile of human breast milk. Immunol. Cell Biol..

[17] Rodewald R., Kraehenbuhl J.P. (1984). Receptor-mediated transport of IgG. J. Cell Biol..

[18] Hurley W.L., Theil P.K. (2011). Perspectives on immunoglobulins in colostrum and milk. Nutrients.

[19] Bourne F.J., Curtis J. (1973). The transfer of immunoglobins IgG, IgA and IgM from serum to colostrum and milk in the sow. Immunology.

[20] Stirling C.M.A., Charleston B., Takamatsu H., Claypool S., Lencer W., Blumberg R.S., Wileman T.E. (2005). Characterization of the porcine neonatal Fc receptor—Potential use for trans-epithelial protein delivery. Immunology.

[21] Cervenak J., Kacskovics I. (2009). The neonatal Fc receptor plays a crucial role in the metabolism of IgG in livestock animals. Vet. Immunol. Immunopathol..

[22] Snoeck V., Peters I.R., Cox E. (2006). The IgA system: A comparison of structure and function in different species. Vet. Res..

[23] Möller R., Hansen G.H., Danielsen E.M. (2017). IgG trafficking in the adult pig small intestine: One- or bidirectional transfer across the enterocyte brush border?. Histochem. Cell Biol..

[24] Guzman-Bautista E.R., Ramirez-Estudillo M.C., Rojas-Gomez O.I., Vega-Lopez M.A. (2015). Tracheal and bronchial polymeric immunoglobulin secretory immune system (PISIS) development in a porcine model. Dev. Comp. Immunol..

[25] Le Jan C. (1996). Cellular components of mammary secretions and neonatal immunity: A review. Vet. Res..

[26] Tuboly S., Bernath S., Glavits R., Medveczky I. (1988). Intestinal absorption of colostral lymphoid cells in newborn piglets. Vet. Immunol. Immunopathol..

[27] Tizard I.R. (2004). Veterinary Immunology: An Introduction.

[28] Tanaka Y., Morita C.T., Tanaka Y., Nieves E., Brenner M.B., Bloom B.R. (1995). Natural and synthetic non-peptide antigens recognized by human γδ T cells. Nature.

[29] Luissint A.C., Parkos C.A., Nusrat A. (2016). Inflammation and the intestinal barrier: Leukocyte–epithelial cell interactions, cell junction remodeling, and mucosal repair. Gastroenterology.

[30] Cabinian A., Sinsimer D., Tang M., Zumba O., Mehta H., Toma A., Sant’Angelo D., Laouar Y., Laouar A. (2016). Transfer of maternal immune cells by breastfeeding: Maternal cytotoxic T lymphocytes present in breast milk localize in the peyer’s patches of the nursed infant. PLoS ONE.

[31] Schlesinger L., Muñoz C., Arevalo M., Arredondo S., Mendez G. (1989). Functional capacity of colostral leukocytes from women delivering prematurely. J. Pediatr. Gastroenterol. Nutr..

[32] Food and Agriculture Organization of the United Nations (FAO) (2017). Food Outlook: Biannual Report on Global Food Markets.

[33] Pappas G. (2013). Socio-economic, industrial and cultural parameters of pig-borne Infections. Clin. Microbiol. Infect..

[34] Rocadembosch J., Amador J., Bernaus J., Font J., Fraile L. (2016). Production parameters and pig production cost: Temporal evolution 2010–2014. Porcine Health Manag..

[35] Jafari A. (2012). *Escherichia coli*: A brief review of diarrheagenic pathotypes and their role in diarrheal diseases in Iran. Iran. J. Microbiol..

[36] Viviana T.L., Alicia Zon M., García Ovando H., Roberto Vettorazzi N., Javier Arévalo F., Fernández H. (2017). Electrochemical Magneto Immunosensor Based on Endogenous B-Galactosidase Enzyme to Determine Enterotoxicogenic *Escherichia coli* F4 (K88) In Swine Feces Using Square Wave Voltammetry. Talanta.

[37] Luppi A., Gibellini M., Gin T., Vangroenweghe F., Vandenbroucke V., Bauerfeind R., Bonilauri P., Labarque G., Hidalgo Á. (2016). Prevalence of virulence factors in enterotoxigenic *Escherichia coli* isolated from pigs with post-weaning diarrhoea in Europe. Porcine Health Manag..

[38] Luppi A. (2017). Swine enteric colibacillosis: Diagnosis, therapy and antimicrobial resistance. Porcine Health Manag..

[39] Amezcua R., Friendship R.M., Dewey C.E., Gyles C., Fairbrother J.M. (2002). Presentation of postweaning *Escherichia coli* diarrhea in southern Ontario, prevalence of hemolytic *E. coli* serogroups involved, and their antimicrobial resistance patterns. Can. J. Vet. Res..

[40] Shahriar F., Ngeleka M., Gordon J., Simko E. (2006). Identification by mass spectroscopy of F4ac-fimbrial-binding proteins in porcine milk and characterization of lactadherin as an inhibitor of F4ac-positive *Escherichia coli* attachment to intestinal villi in vitro. Dev. Comp. Immunol..

[41] Niewold T.A., van Dijk A.J., Geenen P.L., Roodink H., Margry R., van der Meulen J. (2007). Dietary specific antibodies in spray-dried immune plasma prevent enterotoxigenic *Escherichia coli* F4 (ETEC) post weaning diarrhoea in piglets. Vet. Microbiol..

[42] Pereira D., Silva C., Ono M., Vidotto O., Vidotto M. (2015). Humoral Immune Response of Immunized Sows with Recombinant Proteins of Enterotoxigenic *Escherichia coli*. World J. Vaccines.

[43] Fleckenstein J.M., Sheikh A., Qadri F. (2014). Novel antigens for enterotoxigenic *Escherichia coli* vaccines. Expert Rev. Vaccines.

[44] Gamazo C., Martín-Arbella N., Brotons A., Camacho A., Irache J. (2015). Mimicking microbial strategies for the design of mucus-permeating nanoparticles for oral immunization. Eur. J. Pharm. Biopharm..

[45] Kumar P., Luo Q., Vickers T., Sheikh A., Lewis W., Fleckenstein J., Payne S. (2013). Eata, an immunogenic protective antigen of enterotoxigenic *Escherichia coli*, degrades intestinal mucin. Infect. Immun..

[46] Luo Q., Vickers T., Fleckenstein J. (2016). Immunogenicity and protective efficacy against enterotoxigenic *Escherichia coli* colonization following intradermal, sublingual, or oral vaccination with Etpa adhesin. Clin. Vaccine Immunol..

[47] Roy K., Bartels S., Qadri F., Fleckenstein J. (2010). Enterotoxigenic *Escherichia coli* Elicits Immune Responses to Multiple Surface Proteins. Infect. Immun..

[48] Roy K., Hamilton D., Ostmann M.M., Fleckenstein J.M. (2009). Vaccination with EtpA glycoprotein or flagellin protects against colonization with enterotoxigenic *Escherichia coli* in a murine model. Vaccine.

[49] Roy K., Hilliard G., Hamilton D., Luo J., Ostmann M., Fleckenstein J. (2008). Enterotoxigenic *Escherichia coli* Etpa Mediates Adhesion Between Flagella and Host Cells. Nature.

[50] Sheikh A., Luo Q., Roy K., Shabaan S., Kumar P., Qadri F., Fleckenstein J.M. (2014). Contribution of the Highly Conserved EaeH Surface Protein to Enterotoxigenic *Escherichia coli* Pathogenesis. Infect. Immun..

[51] Van den Broeck W., Cox E., Oudega B., Goddeeris B. (2000). The F4 Fimbrial Antigen of *Escherichia coli* and Its Receptors. Vet. Microbiol..

[52] Verdonck F. (2004). Oral Immunization of Piglets with Recombinant F4 Fimbrial Adhesin Faeg Monomers Induces a Mucosal and Systemic F4-Specific Immune Response. Vaccine.

[53] Nagy B., Fekete P. (2005). Enterotoxigenic *Escherichia coli* in Veterinary Medicine. Int. J. Med. Microbiol..

[54] Pereira D., Vidotto M., Nascimento K., Santos A., Mechler M., Oliveira L. (2016). Virulence Factors of *Escherichia coli* In Relation To The Importance Of Vaccination In Pigs. Ciência. Rural.

[55] Azegami T., Yuki Y., Kiyono H. (2014). Challenges in mucosal vaccines for the control of infectious diseases. Int. Immunol..

[56] Rodriguez-Pineiro A.M., Bergstrom J.H., Ermund A., Gustafsson J.K., Schutte A., Johansson M.E.V., Hansson G.C. (2013). Studies of mucus in mouse stomach, small intestine, and colon. II. Gastrointestinal mucus proteome reveals Muc2 and Muc5ac accompanied by a set of core proteins. Am. J. Physiol. Gastrointest. Liver Physiol..

[57] Srivastava A., Gowda D.V., Madhunapantula S.V., Shinde C.G., Iyer M. (2015). Mucosal vaccines: A paradigm shift in the development of mucosal adjuvants and delivery vehicles. APMIS.

[58] Salmon H. (2003). Immunophysiology of the mammary gland and transmission of immunity to the young. Reprod. Nutr. Dev..

[59] Vela Ramirez J.E., Sharpe L.A., Peppas N.A. (2017). Current state and challenges in developing oral vaccines. Adv. Drug Deliv. Rev..

[60] Di Pasquale A., Preiss S., Tavares da Silva F., Garçon N. (2015). Vaccine adjuvants: From 1920 to 2015 and beyond. Vaccines.

[61] Keller-Stanislawski B., Englund J.A., Kang G., Mangtani P., Neuzil K., Nohynek H., Pless R., Lambach P., Zuber P. (2014). Safety of immunization during pregnancy: A review of the evidence of selected inactivated and live attenuated vaccines. Vaccine.

[62] Walker R.I. (2015). An assessment of enterotoxigenic *Escherichia coli* and *Shigella* vaccine candidates for infants and children. Vaccine.

[63] Fairbrothera J.M., Nadeau E., Bélanger L., Tremblay C.L., Tremblay D., Brunelle M., Wolf R., Hellmann K., Hidalgo A. (2017). Immunogenicity and protective efficacy of a single-dose live non-pathogenic *Escherichia coli* oral vaccine against F4-positive enterotoxigenic *Escherichia coli* challenge in pigs. Vaccine.

[64] Nadeau É., Fairbrother J.M., Zentek J., Bélanger L., Tremblay D., Tremblay C.L., Röhe I., Vahjen W., Brunelle M., Hellmann K. (2017). Efficacy of a single oral dose of a live bivalent *E. coli* vaccine against post-weaning diarrhea due to F4 and F18-positive enterotoxigenic *E. coli*. Vet. J..

[65] Cox E., Melkebeek V., Devriendt B., Goddeeris B., Vanrompay D., Stefano M. (2014). Vaccines against enteric *E. coli* infections in animals. Pathogenic Escherichia coli: Molecular and Cellular Microbiology.

[66] Baxter D. (2007). Active and Passive Immunity, Vaccine Types, Excipients and Licensing. Occup. Med..

[67] Lee H., Choi J. (2017). Tetanus–Diphtheria–Acellular Pertussis Vaccination for Adults: An Update. Clin. Exp. Vaccine Res..

[68] Chong C., Friberg M., Clements J. (1998). LT(R192G), A non-toxic mutant of the heat-labile enterotoxin of *Escherichia coli*, elicits enhanced humoral and cellular immune responses associated with protection against lethal oral challenge with *Salmonella* Spp.. Vaccine.

[69] Zhang W., Zhang C., Francis D., Fang Y., Knudsen D., Nataro J., Robertson D. (2009). Genetic fusions of heat-labile (LT) and heat-stable (ST) toxoids of porcine enterotoxigenic *Escherichia coli* elicit neutralizing anti-LT and anti-STA antibodies. Infect. Immun..

[70] Snoeck V., Huyghebaert N., Cox E., Vermeire A., Vancaeneghem S., Remon J., Goddeeris B. (2003). Enteric-coated pellets of F4 fimbriae for oral vaccination of suckling piglets against enterotoxigenic *Escherichia coli* infections. Vet. Immunol. Immunopathol..

[71] Irache J.M., Esparza I., Gamazo C., Agüeros M., Espuelas S. (2011). Nanomedicine: Novel approaches in human and veterinary therapeutics. Vet. Parasitol..

[72] Gamazo C., Gastaminza G., Ferrer M., Sanz M.L., Irache J.M. (2014). Nanoparticle based-immunotherapy against allergy. Immunotherapy.

[73] Gamazo C., Irache J.M., Alonso M.J., Csaba N.S. (2012). Nanostructures for oral vaccine delivery. Nanostructured Biomaterials for Overcoming Biological Barriers.

[74] Kim S., Doh H., Jang M., Ha Y., Chung S., Park H. (1999). Oral Immunization With *Helicobacter Pylori*-Loaded Poly(d,l-Lactide-*co*-Glycolide) Nanoparticles. Helicobacter.

[75] Porporatto C. (2005). Local and Systemic Activity of the Polysaccharide Chitosan at Lymphoid Tissues after Oral Administration. J. Leukoc. Biol..

[76] Petersen L., Ramer-Tait A., Broderick S., Kong C., Ulery B., Rajan K., Wannemuehler M., Narasimhan B. (2011). Activation of innate immune responses in a pathogen-mimicking manner by amphiphilic polyanhydride nanoparticle adjuvants. Biomaterials.

[77] Ulery B., Kumar D., Ramer-Tait A., Metzger D., Wannemuehler M., Narasimhan B. (2011). Design of a protective single-dose intranasal nanoparticle-based vaccine platform for respiratory infectious diseases. PLoS ONE.

[78] Felder C., Vorlaender N., Gander B., Merkle H., Bertschinger H. (2000). Microencapsulated enterotoxigenic *Escherichia coli* and detached fimbriae for peroral vaccination of pigs. Vaccine.

[79] Tamayo I., Irache J.M.I., Mansilla C., Ochoa-Reparaz J., Lasarte J., Gamazo C. (2010). Poly(Anhydride) nanoparticles act as active Th1 adjuvants through toll-like receptor exploitation. Clin. Vaccine Immunol..

[80] Camacho A., Da Costa Martins R., Tamayo I., de Souza J., Lasarte J., Mansilla C., Esparza I., Irache J., Gamazo C. (2011). Poly(Methyl Vinyl Ether-Co-Maleic Anhydride) nanoparticles as innate immune system activators. Vaccine.

[81] Vandamme K., Melkebeek V., Cox E., Remon J., Vervaet C. (2011). Erratum to “Adjuvant Effect of Gantrez^®^AN Nanoparticles during oral vaccination of piglets against F4+enterotoxigenic *Escherichia coli*. Vet. Immunol. Immunopathol..

[82] Salman H., Irache J., Gamazo C. (2009). Immunoadjuvant Capacity of Flagellin And Mannosamine-Coated Poly(Anhydride) Nanoparticles in oral vaccination. Vaccine.

[83] Jose Matías J., Lasierra T., Pérez-Guzmán I., Cenoz S., Irache J.M.I., Gamazo C. (2016). Nanoparticles Formulated from Protein Food-Born Polymers in the Development of a Mucosal Complex Vaccine against ETEC.

[84] Davis H. (2008). Novel vaccines and adjuvant systems: The utility of animal models for predicting immunogenicity in humans. Hum. Vaccine.

